# *eBryoSoil*: a citizen science application to monitor changes in soil ecosystems

**DOI:** 10.1038/s41598-024-74464-8

**Published:** 2024-10-19

**Authors:** André F. Mira, Joaquín Hortal, Ana Paula Portela, Belén Albertos, Belén Estébanez, Cristina Branquinho, Cristiana Vieira, Helena Hespanhol, Isabel Draper, Joana Marques, Juliana Monteiro, María Leo, Pilar Hurtado, Raúl Ochoa-Hueso, Zulema Varela, Nagore G. Medina

**Affiliations:** 1 Department Biogeography and Global Change, Museo Nacional de Ciencias Naturales (MNCN-CSIC), Madrid, 28006 Spain; 2 Department of Biology, Universidad Autónoma de Madrid, Madrid, 28049 Spain; 3 Programa de Doctorado en Conservación de Recursos Naturales, Universidad Rey Juan Carlos, Madrid, 28933 Spain; 4 Centre for Ecology, Evolution and Environmental Changes (cE3c) & Global Change and Sustainability Institute (CHANGE), Faculdade de Ciências, Universidade de Lisboa, Lisbon, 1749-016 Portugal; 5 Centro de Investigação em Biodiversidade e Recursos Genéticos, CIBIO, InBIO Laboratório Associado, Universidade do Porto, Campus de Vairão, Vairão, 4485-661 Portugal; 6 Department of Biology, Faculdade de Ciências, Universidade do Porto, Porto, 4169– 007 Portugal; 7 BIOPOLIS Program in Genomics, Biodiversity and Land Planning, CIBIO, Vairão, 4485-661 Portugal; 8 Department de Botànica i Geologia, Universitat de València, Burjassot, 46100 Spain; 9 Museu de História Natural e da Ciência da Universidade do Porto (MHNC-UP/UPorto/PRISC), Porto, 4099-002 Portugal; 10 Centro de Investigación en Biodiversidad y Cambio Global (CIBC-UAM), Universidad Autónoma de Madrid, Madrid, 28049 Spain; 11 Department Soil, Plant and Environmental Quality, Instituto de Ciencias Agrarias (ICA-CSIC), Madrid, 28006 Spain; 12 Biodiversity and Conservation Area, Universidad Rey Juan Carlos, Madrid, 28933 Spain; 13 DIFAR, University of Genoa, Genoa, 16148 Italy; 14 Department of Biology, IVAGRO, Universidad de Cádiz, Campus of International Agri-Food Excellence (ceiA3), Cádiz, 11510 Spain; 15 Ecology Unit, Department of Functional Biology, CRETUS, Universidade de Santiago de Compostela, Santiago de Compostela, 15872 Spain

**Keywords:** Biological soil covers (BSCs), Citizen science, Climate change, Iberian Peninsula, Soil communities, eBryoSoil, Ecology, Ecology, Environmental sciences

## Abstract

Biological soil covers (BSCs) play a pivotal role in ecosystem functioning by enhancing soil stability, mediating nutrient cycling, and influencing soil hydrology. Recognized as ecosystem engineers, they can physically modify, maintain, or create habitats, facilitating plant community development. Through these intricate interactions, BSCs contribute significantly to ecological processes, highlighting their importance in the overall health and functionality of the ecosystems of the Iberian Peninsula. Here we present the results obtained from the contributions of the citizen scientists uploaded from November 2019 to January 2021 with *eBryoSoil*, an app that allows citizens to participate in mapping the BSC communities across the Iberian Peninsula. Here, we emphasize the importance of habitats and consequently, their interaction with climatic variables for the persistence of BSCs (lichens and bryophytes). Conservation efforts targeted at preserving diverse habitats are essential to ensure the continued presence of lichen and bryophyte communities. Despite challenges posed by the SARS-CoV-2 outbreak, this citizen science project demonstrated success in utilizing a specifically tailored app to gather valuable information on BSC communities, providing insights into their vulnerability to climate change. This program serves as an illustrative example of how citizen science can effectively identify and study vulnerable habitats, offering a blueprint for future studies focused on understudied organisms.

## Introduction

Biological soil covers (BSCs) are soil-dwelling communities composed of photosynthetic organisms such as lichens, bryophytes, and microscopic algae living in close association with bacteria, fungi, protists, and microarthropods such as springtails. These communities play an essential role in ecosystem functioning, enhancing soil stability^1–3^, mediating nutrient cycling^4–6^, influencing soil hydrology^7^and promoting plant community development^6,8^. Thus, BSCs act as ecosystem engineers, because they can physically modify, maintain, and/or create new habitats that make the conditions more suitable for other species^1,9^.

Many of the species that make up BSCs are facing increased vulnerability due to global change^10–12^. In particular, climate change is altering precipitation patterns and inducing more frequent droughts across many regions. Among the ecosystems significantly affected are drylands, which are naturally water limited. In these ecosystems, BSCs cover up to 70% of the surface and provide crucial contributions to primary production and nitrogen fixation^5,13–15^, making them particularly vulnerable to changes in the climatic regime^16^. Such vulnerability is largely due to the poikilohydric nature of most components of BSCs, meaning they are only metabolically active when they are wet^17^, to a point that the frequency of precipitation can be used as a proxy of their presence and composition^7^, at least in dry environments. Furthermore, BSCs are also sensitive to other climate change-related parameters. For instance, studies have demonstrated that large temperature variations cause changes in the composition of the cyanobacterial populations, which constitute the bulk of the biomass in dryland BSCs^13,18^. Besides, an increase in temperature usually correlates negatively with hydration in dry climates, and thus restricts the metabolic activity of the organisms that form BSCs^19^. Additionally, constituents of BSCs may experience different effects. Bryophytes are usually very sensitive to high temperatures in a hydrated state, but tolerant to them when dry^20,21^. On the other hand, lichens are more sensitive to desiccation damage, which may result in a reduction of their photosynthetic activity^22,23^. Furthermore, the recovery of these communities is slow, as their water-acquisition dynamics lead to low productivity levels resulting in long recovery times that span several hundreds of years^24^. Even though we have relatively detailed information about the vulnerability of BSCs to climate change in drylands, information for other ecosystem types is more limited. Consequently, it is still unclear how BSCs will continue to respond to changing climatic conditions across various habitats and ecosystems.

Given the limited understanding of BSC responses across different ecosystems, and the alarming rate at which climate change is affecting natural systems^25–27^there is a pressing need to accelerate the pace of knowledge acquisition. Effective conservation planning requires detailed data on the distribution of biodiversity and its responses to these long-term threats. Moreover, the available information may rapidly become obsolete, as the rates of distributional shift of the diverse species forming BSCs in response to climate change is also highly variable^28^. Despite the urgent need for high-quality data, generating such information can be time-consuming and resource-intensive, thus posing a significant challenge. This is especially true for small-sized and inconspicuous organisms such as those composing BSCs, neglected by both the public and the researchers, so that they have not received recognition and efforts matching the significance of their contributions to ecosystem functioning^29^.

The shortage of data about biodiversity highlights the need for innovative and effective strategies to improve the quantity, quality, and integrity of the obtained information^30^. Smartphone-based citizen science projects can help to reduce data gaps by combining the reach of public volunteers with current smartphone technology to generate large scientific datasets^31^. These types of projects offer an excellent opportunity to overcome many of the challenges faced by researchers, such as time, reach, and/or human resources^32^. Citizen science programs often face the need for simple data collection protocols^33^, resulting in a preferential focus on flagship organisms that are charismatic and/or easily identifiable^34^. This approach reinforces the existing bias towards large organisms, leaving many groups such as BSCs unexplored. Yet, most of the world’s biodiversity is concentrated in small-sized organisms that are difficult to identify. Therefore, developing programs targeting these organisms that raise awareness about their relevance should be a key goal for citizen science in the coming decades. For instance, several projects such as ‘*Soils for Science -*https://imb.uq.edu.au/soilsforscience/*’* and ‘*Earthworm Watch -*https://www.earthwormwatch.org/’ (for more soil-focused projects see^35^) have already shown how the design of citizen-science initiatives specifically tailored to study small or neglected organisms can engage the general public into their conservation, while providing key data for biodiversity research. Moreover, it is not only research that benefits from such endeavors; these activities constitute a reciprocal exchange, whereby society also gains increased awareness of biodiversity conservation and environmental issues^36^.

In this paper we aim to assess the potential value of citizen science to study BSCs dominated by bryophytes, lichens, and cyanobacteria. *SoilSkin* citizen science project intends to expand knowledge of Iberian soil ecosystems in the face of climate change by training the public to identify BSC communities and quantify their coverage. This project combines educational activities with the specifically tailored mobile app *eBryoSoil* for data collection, aiming to fill the knowledge gap on BSCs distribution. Specifically, our objectives are: (i) testing the performance of a smartphone app-based citizen science program (*SoilSkin*) on a group of organisms that is scarcely known by most citizens, and (ii) increasing the information on the distribution and vulnerability of BSCs to disturbances. To do this, we analyze the data collected during a pilot study conducted mostly by researchers over 14 months, to assess the validity of this approach for monitoring the responses of BSCs at large scales.

## Materials and methods

### *eBryoSoil *smartphone app

*eBryoSoil* is a free smart-device Android application developed using Java and connected to a PHP database in an external server. This app was launched in 2019 by *eBryo* research group on experimental bryology. *eBryoSoil* runs on Android platforms and only requires a mobile device with a compatible Android 4.4 Operating System or superior, a rear camera, and a GPS to provide information on location. Citizens can autonomously gather information about the coverage of BSCs using this app, although it does require some basic training, as the users need to identify the types of BSCs and of their visible organisms and assess their relative cover in the soil. Due to this, the app is coupled with *SoilSkin*, a program of educational activities that includes such training (see https://ebryo.com/soilskin/*).*

The protocol of sampling BSC communities with the *eBryoSoil* app involves taking three photographs of the soil communities spaced 10 m along a 30-meter transect. The user must find an area where BSCs are present. A transect starts when the user finds a suitable BSC community and takes a photograph of it. The latter is always taken at the same distance from the soil, using a coin as a reference. Although the precise size of the sampled area may vary from one smartphone device to another, the area covered by each photograph is approximately 22 × 12 cm. After the acquisition of the image, users must quantify the percentage cover of the different types of BSCs present in the image. For that purpose, we generated a simplified soil cover classification based on BSC types and growth forms (Table 1; for more information regarding classification^12^, see^37^for lichens and^38^ for bryophytes). Users must then walk 15 m in a straight line to do a second record, and 15 m more for the last one. The users also must select the type of habitat in which the three photographs of the transect were taken, from a list of seven possible habitats (Table 1). The transect is complete when all three pictures and their respective information are uploaded, which are collected simultaneously with a GPS location and a time stamp. Data from each transect is finally uploaded and stored on an external server, either at the moment of collection or later if the mobile data connection is poor, which allows to gather records even in remote areas. A detailed description of the protocol is available at https://ebryo.com/download/279/.

**Table 1:** Categories recorded by users for each record in *eBryoSoil* smartphone app. Soil cover is recorded for each picture, as percentages within the photograph; lichen and bryophyte cover (in italics) correspond to the sum of percentages of their growth forms. Habitat type is recorded once per transect (see main text).

Biological Soil Cover type	Habitat type
*Lichen*	Open Forest
Foliose	Dense Forest
Fruticose	Shrubland
*Bryophyte*	Grassland
Acrocarpous	Urban Green Spaces
Pleurocarpous	Agricultural Land
Crustose	Coastal
Algae	
Bare soil	

### Data collection and quality assurance

For the present study, we only considered the records submitted between 26/11/2019 and 31/1/2021. Since all records done by citizens were accompanied by a photograph, it was possible to verify all the data uploaded. Thus, all the photographs were visualized, and we corrected the data whenever we detected a mismatch between the cover percentage described and the cover on the photograph. We performed further corrections of the habitat type of the collected data using the Q-Geographic Information System (QGIS) software^39^(version 3.16.1). A QGIS project was created with the maps made available as part of the Global Administrative Areas (GADM) plugin^40^ and the coordinates of the records (Fig. 2). This allowed validating the habitat where the records uploaded by the users were obtained. All records that did not require correction and the ones that were corrected were considered valid records. The remaining records were excluded from the analysis as it was not possible to confirm the accuracy of the data (e.g., records with blurred photographs, with no GPS coordinates). The records used in this article are available at^41^.

## Environmental predictors

We obtained 19 bioclimate variables from WorldClim version 2.1 climate data for 1970–2000^42^  at a spatial resolution of 30 arcsec (~ 1 km^2^). These variables represent different aspects of climate, including annual averages (e.g., mean annual temperature, annual precipitation), seasonality (e.g., annual range in temperature and precipitation) and extreme or limiting conditions (e.g., temperature of the coldest and warmest month, and precipitation of the wet and dry quarters). We extracted the values of these 19 environmental variables for the coordinates of each record using QGIS.

## Statistical analyses

In the first segment of the analysis, we used all BSC types to select which bioclimatic variables had a greater impact in their presence/cover. To do so, we used redundancy analysis^43^. This ordination method shows how the variation in the cover of the different types of the BSCs (Inertia) can be explained by the matrix of explanatory variables (Constrained Inertia). If the output of the model was significant, we selected the significant predictors using a forward-selection procedure^44^followed by a Monte Carlo permutation test with a Bonferroni-type adjustment for significance levels^45–47^. From the 19 climatic variables tested, two variables were selected to advance into the next step of the analysis: precipitation of the warmest quarter and minimum temperature of the coldest month.

Apparent difficulties in the convergence of the models explained below suggested that analyzing each type of BSCs individually would not be the best fit for the current data, given the unequal frequency of some BSC types. As a solution for the analysis, a single and new response variable was generated – total lichen and bryophyte cover – which corresponds to the aggregate cover of all bryophytes (acrocarpous and pleurocarpous) and lichens (foliose, fruticose, and crustose) types found in each record. Furthermore, all records set in agricultural land and coastal habitats had to be removed from the analysis due to their low numbers of records (9 and 17 records, respectively). Since the response variable was proportional and contained several occurrences of zero (0% cover) and one (100% cover) value, we used a Bayesian inference model for beta regression and zero/one inflated beta regression, using the R-package ‘zoib’^48^. The main function of ‘zoib’ uses Bayesian inference with Markov Chain Monte Carlo sampling to simultaneously estimate the mean (linear predictor for values between 0 and 1), the probability of zero, and the probability of one. However, while this package can deal with zero and one inflated data, the low number of records that quantified 100% cover did not justify the use of the one inflated component of the model, and so, all records with 100% cover were transformed to 99.9% cover. All zero-inflated beta regression candidate models (with different factor combinations, using ‘*location’* or ‘*transect*’ as a random factor) were run with two Markov Chain Monte Carlo chains for 20,000 iterations, a burn-in period of 2,000, and a thinning of 40. To confirm the convergence of the chains, an inspection of the potential scale reduction factor values and trace plot of each model were performed. The final model was chosen among all candidate models by a comparison using their Deviance Information Criterion (DIC) values^49,50^.

## Results

### User engagement and survey performance

**Fig. 1:**
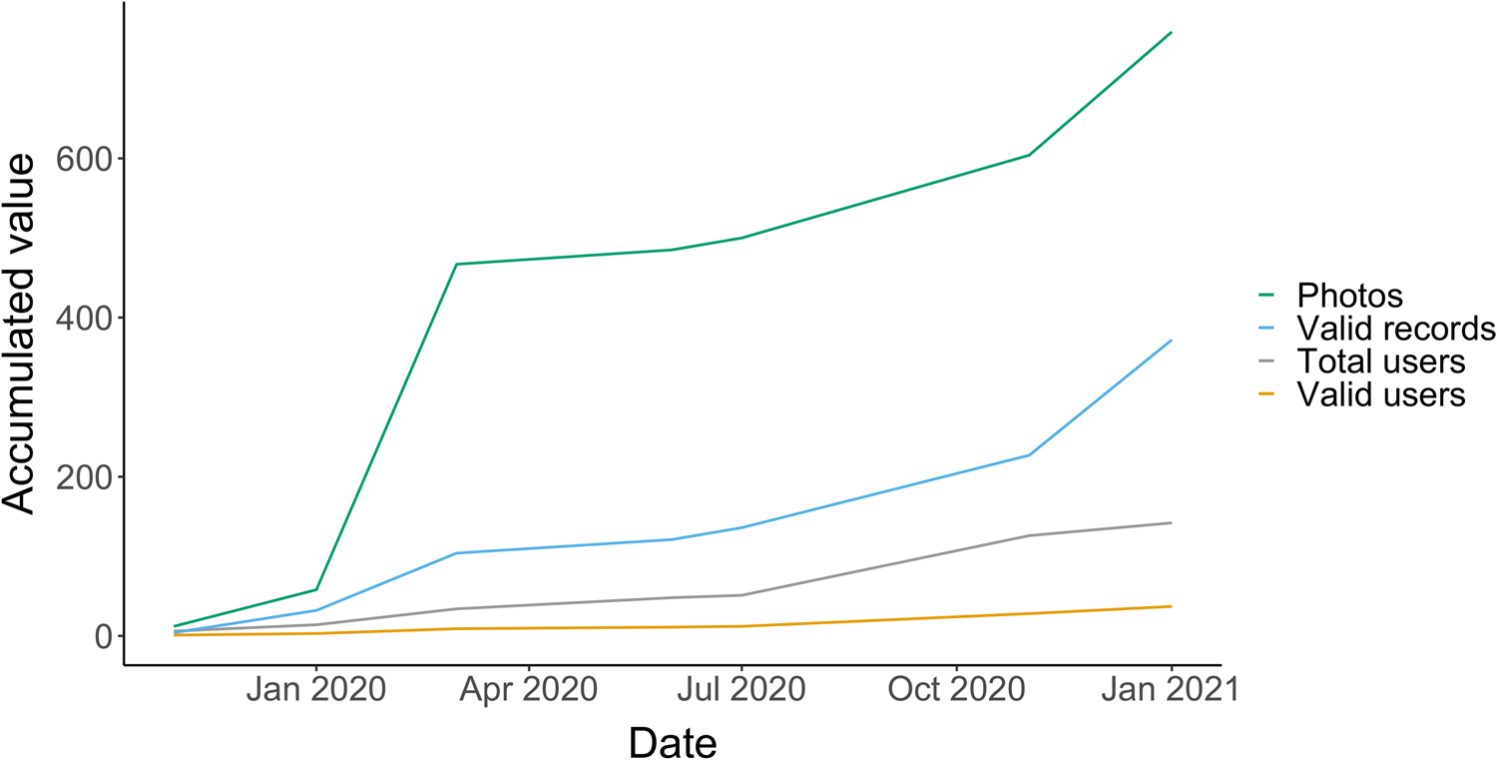
Accumulated number of total records (dark green, first line from the top), valid records (light blue, second line from top), total users (light grey, third line from the top) and users with valid records (orange, fourth line from the top) during the first 14 months after the release of the eBryoSoil mobile app.

From 2019 to 2021, the *SoilSkin* citizen science project organized more than 30 workshops, with a total of 800 participants. During these workshops, attendees learned about the importance of BSCs and how they could contribute to increase our knowledge of them through citizen science surveys using the *eBryoSoil* mobile app. The high attendance at the workshops contributed significantly to the number of users registered in the mobile app; 142 unique users registered between 26/11/2019 and 31/1/2021. The number of registered users and uploads on the mobile app increased throughout the study (Fig. 1). However, we identified two periods of reduced activity: from March 2020 to October 2020, and from December 2020 onwards. These periods coincide with the outbreak of the SARS-CoV-2 pandemic, and the two most restrictive implementations of population confinement measures in the Iberian Peninsula.

The users uploaded a total of 759 photographs. For the present study, we analyzed a selection of 372 records with valid information (49% of all records in the database), which were uploaded by 37 users (Fig. 1).

The average number of valid uploads by user was approximately 9 records, while the most common number of valid uploads was 3 (11 times). Furthermore, the 5 highest contributors presented a total of 48% of the valid uploads (178 valid uploads), while out of the 40 users with valid records, only 6 users were responsible for uploading 1 valid record (1.6%).

### Location and quality of the records

**Fig. 2:**
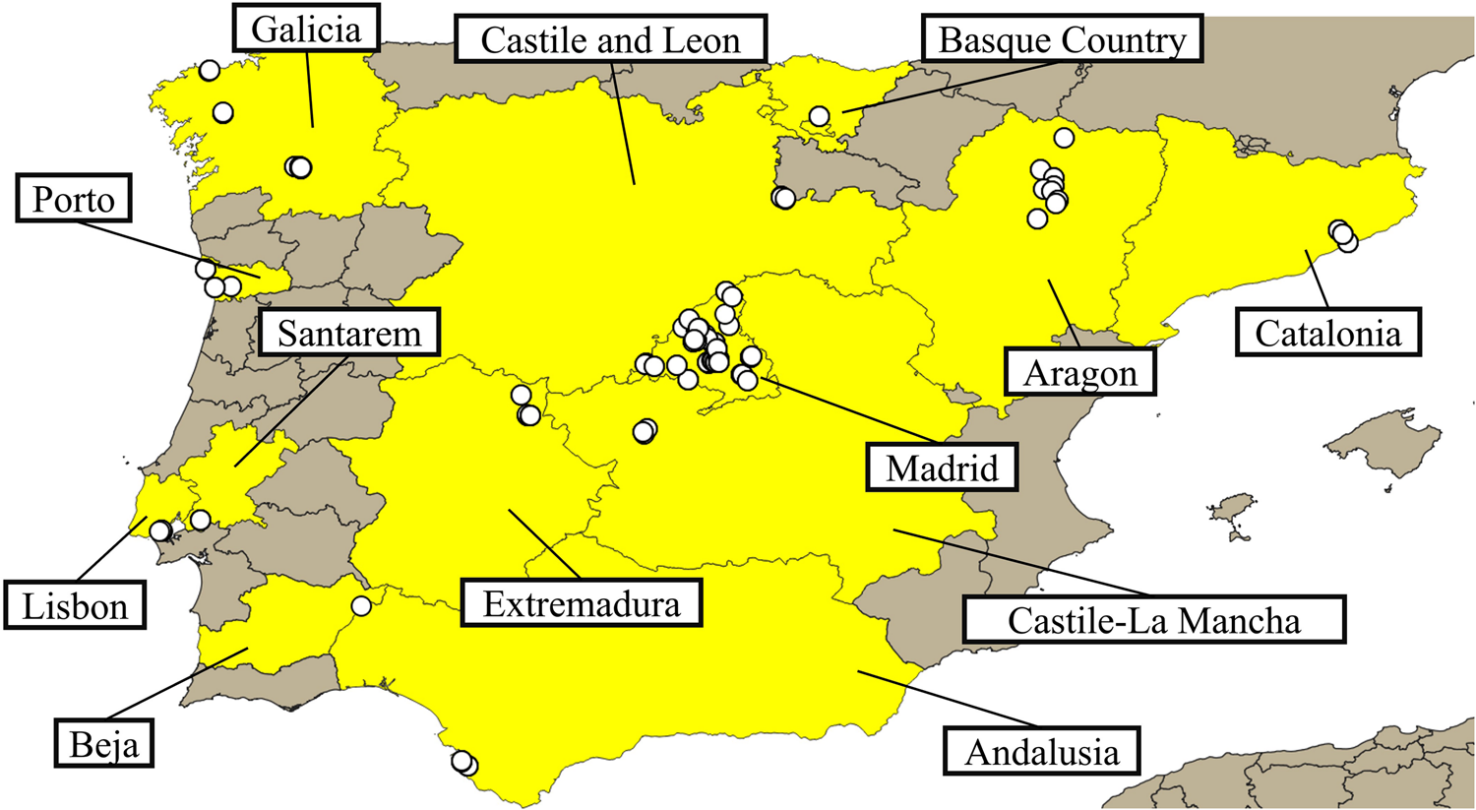
Location of valid records by the users of the eBryoSoil mobile app around the Iberian Peninsula. Regions with records are presented in yellow. Map generated in QGIS^39^ (version 3.16.1, https://www.qgis.org/).

The *eBryoSoil* mobile app was used in 13 administrative units across the Iberian Peninsula (Fig. 2). Madrid had the highest number of valid records with 175 records (47.04%), followed by Galicia and Catalonia with 36 (9.7%) and 33 (8.9%) records, respectively. Four other regions had more than 5% of valid records including Extremadura with 27 records (7.2%), Aragon with 22 (5.9%), and Porto and Andalusia with 19 (5.1%). The remaining six regions had less than 5% of the valid records, Lisbon having 15 registers (4,1%), followed by Castile and Leon with 9 records (2.4%), Extremadura and Castile – La Mancha with 8 (2.2%), Basque Country with 6 (2.1%), Santarem with 2 (0.4%), and Beja with 1 valid record (0.2%).

**Fig. 3:**
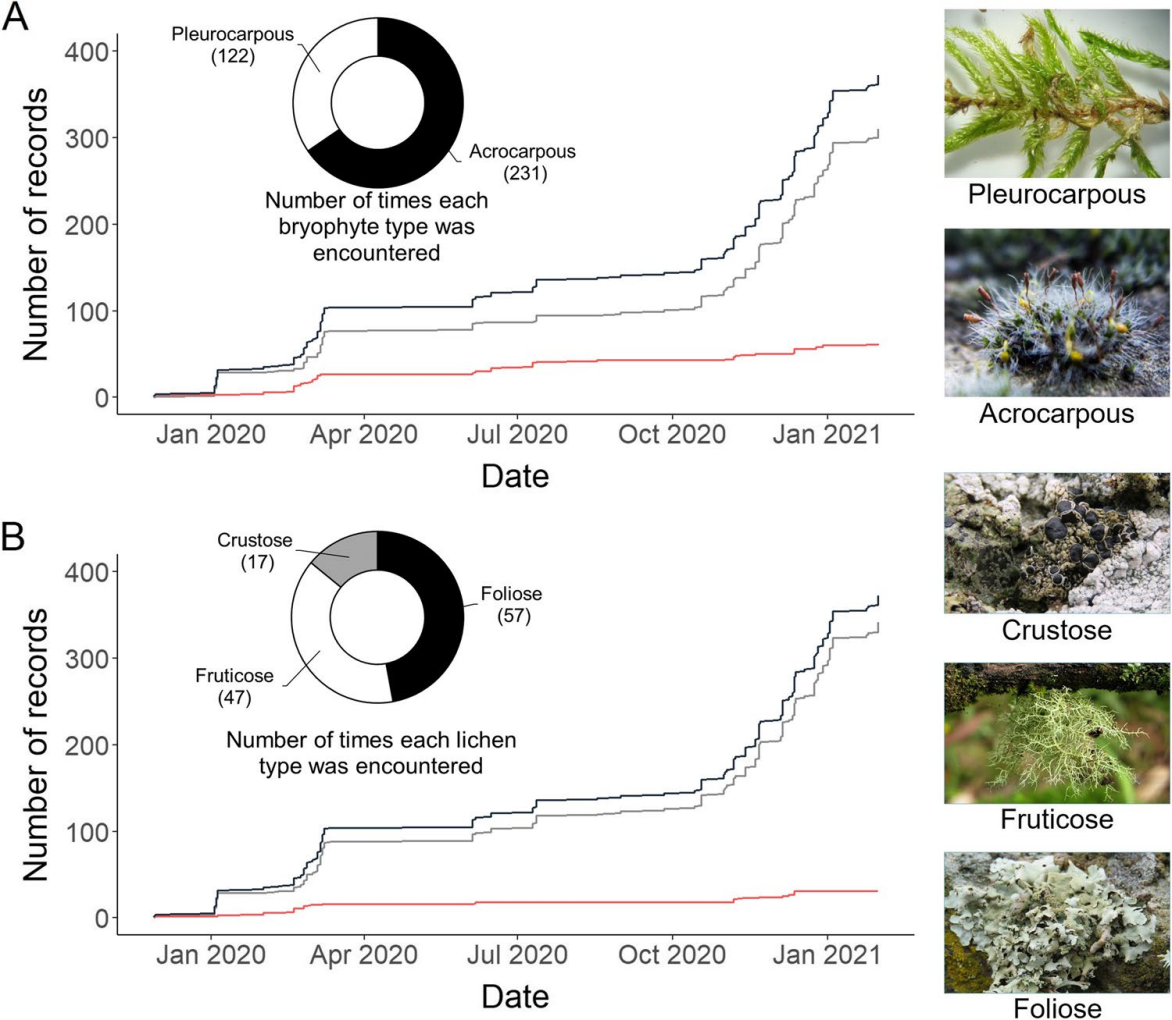
Temporal increment of the data uploaded by users of the eBryoSoil mobile application and their respective performance in the quantification of the cover occupied by two biological soil cover types – bryophytes (**A**) and lichens (**B**). Within each graph, the growth of the total records uploaded (black line) was divided into the number of records that correctly (grey lines) and incorrectly (red line) quantified the cover percentage. Pie charts illustrate the number of times each type of bryophyte or lichen was identified by the users across all the records.

Of the 372 valid records, 78 required a correction for the type of biological soil community identified or the percentage cover quantified (Fig. 3). Bryophytes were identified 329 times, with acrocarpous bryophytes being the most common type observed. Lichens were encountered in 103 records, with foliose and fruticose types being more frequent (Fig. 3). 62 bryophyte records (19%) and 31 lichen records (30%) were deemed invalid and required corrections.

Regarding the habitats, the highest number of valid records was found in open forest habitats, with 104 records (30%), followed by dense forest habitats, with 67 records (18%). The remaining habitats, green urban spaces, shrubland and grasslands represented a total of 66 (17.7%), 60 (16.1%) and 49 (13.1%) of the valid registers, respectively. Moreover, 17 (4.6%) uploaded records were done in agricultural land and 9 (2.4%) in coastal habitats, but as mentioned before, these records were removed from the following analysis.

## Relationships of BSCs coverage with climate and habitat

For the following analysis, BSC refers exclusively to the combined cover of all types of lichens and bryophytes. The variable selection procedure based on redundancy analyses identified two climatic variables (*P* < 0.05) that explained a significant proportion of the variation in the BSC cover: minimum temperature of the coldest month, and precipitation of the warmest quarter. These variables contributed to explain 15.1% and 1.2% of the variance in the first and second constrained axes, respectively.

The zero/one inflated beta regression model that includes ‘transect’ as a random variable, along with the explanatory variables ‘habitat’ and both climatic variables selected by the forward-selection procedure and their respective interaction with ‘habitat’, was selected as the fittest model to the data, and therefore, was used in our analysis (see Table 2). In terms of habitat preferences, our findings indicate that, although BSC presence was similar in both dense forest and open forest habitats, their cover is significantly higher in the dense forest plots. Conversely, BSC cover was lower in shrubland habitats. Moreover, our results revealed that BSCs are more frequently present in grassland habitats than in open forest habitats, but there were no significant differences in their mean cover. In green urban spaces habitats, the presence and cover of BSCs exhibited considerable variability, with no significant differences compared to open forest habitats.

**Table 2:** Posterior inferences of the coefficients (on the logit-scale) in the best bayesian zero-inflated beta distribution model of the total lichen and bryophyte cover. The first model component estimates the mean (linear predictor) in the model, and the second the probability of zero cover. The factor level *open forest* is the baseline value in the model and is included in the intercept. A significant difference is found when the quantile range does not overlap zero.

Model Component	Effect	Estimate	SD	2.5% quantile	97.5% quantile	
logit (mean)	(Intercept)	0.0891	0.3429	-0.5803	0.7240	
	**Dense forest**	**1.0610**	**0.5239**	**0.0434**	**2.0994**	*
	Green urban spaces	0.3065	0.5385	-0.7500	1.3512	
	Grassland	-0.9712	0.5964	-2.1824	0.1822	
	**Shrubland**	**-1.3670**	**0.5613**	**-2.5100**	**-0.2337**	*
	Precipitation of the warmest quarter	0.0035	0.0038	-0.0035	0.0111	
	Minimum temperature of the coldest month	-0.0359	0.0647	-0.1628	0.0904	
	Dense forest x precipitation of the warmest quarter	-0.0086	0.0050	-0.0186	0.0008	
	Green urban spaces x precipitation of the warmest quarter	0.0004	0.0056	-0.0106	0.0112	
	Grassland x precipitation of the warmest quarter	0.0058	0.0053	-0.0047	0.0161	
	**Shrubland x precipitation of the warmest quarter**	**0.0125**	**0.0056**	**0.0016**	**0.0238**	*
	Dense forest x minimum temperature of the coldest month	-0.1142	0.1070	-0.3265	0.0968	
	Green urban spaces x minimum temperature of the coldest month	-0.0981	0.1172	-0.3317	0.1227	
	Grassland x minimum temperature of the coldest month	0.0871	0.1000	-0.1051	0.2847	
	Shrubland x minimum temperature of the coldest month	0.0591	0.1139	-0.1629	0.2839	
logit (Pr (Y = 0))	(Intercept)	0.2703	1.8160	-2.5995	4.3258	
	Dense forest	-2.0840	1.9812	-6.4669	1.4482	
	Green urban spaces	4.1160	4.7284	-2.9472	17.0863	
	**Grassland**	**-17.4800**	**11.8968**	**-47.6784**	**-3.0485**	*
	Shrubland	-0.6043	2.5590	-5.8678	4.3495	
	Precipitation of the warmest quarter	-0.0365	0.0282	-0.1032	0.0051	
	**Minimum temperature of the coldest month**	**-0.6327**	**0.3509**	**-1.4276**	**-0.0883**	*
	Dense forest x precipitation of the warmest quarter	0.0308	0.0290	-0.0149	0.0977	
	Green urban spaces x precipitation of the warmest quarter	-0.0598	0.0841	-0.2975	0.0559	
	**Grassland x precipitation of the warmest quarter**	**0.0918**	**0.0556**	**0.0125**	**0.2195**	*
	Shrubland x precipitation of the warmest quarter	0.0073	0.0389	-0.0694	0.0887	
	Dense forest x minimum temperature of the coldest month	0.6577	0.3934	-0.0129	1.5183	
	Green urban spaces x minimum temperature of the coldest month	-0.3889	0.6376	-1.6838	0.7369	
	**Grassland x minimum temperature of the coldest month**	**2.6920**	**1.3833**	**0.9007**	**6.1287**	*
	Shrubland x minimum temperature of the coldest month	0.3198	0.4456	-0.5080	1.2678	
	**d**	**0.8962**	**0.0940**	**0.7084**	**1.0712**	*
	**σ**	**0.4940**	**0.1513**	**0.2402**	**0.8316**	*

**Fig. 4:**
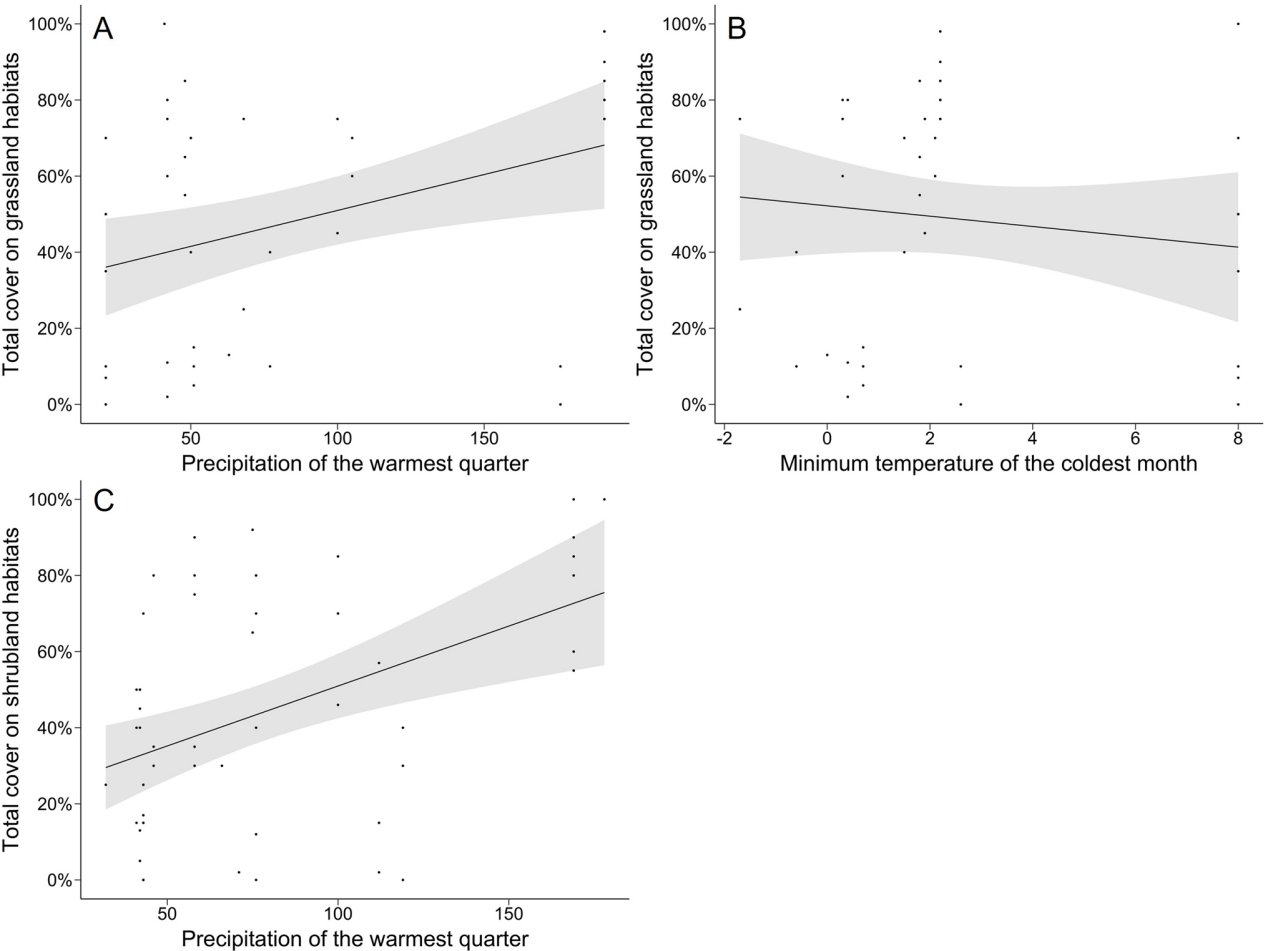
Influence of environmental variables on the total lichen and bryophyte cover present on Grassland habitats: (**A**) Minimum temperature of the coldest month and (**B**) Precipitation of the warmest quarter, and (**C**) Influence of the precipitation of the warmest quarter on the variable total lichen and bryophyte cover present in shrubland habitats. The grey area represents the 95% credible interval.

Regarding climatic variables, the minimum temperature of the coldest month’ exhibited an overall positive effect on the presence of BSCs, which can be seen through the overall negative effect on the probability of records with zero lichen and bryophyte cover. This implies that locations with colder temperatures have a higher likelihood of containing lichen and bryophyte soil communities. In contrast, in grassland habitats, the probability of records with zero lichen and bryophyte cover increased with the interaction with the minimum temperature of the coldest month. Furthermore, a significant positive interaction was observed between precipitation of the warmest quarter and the shrubland habitat (see Fig. 4). Such a result could suggest that higher precipitations might facilitate the development of lichen and bryophyte communities in what normally is a harsh and dry habitat. Finally, both the environmental variable precipitation of the warmest quarter and the grassland habitat had a greater probability of records with zero lichen ​​and bryophyte cover (see Fig. 4).

## Discussion

In this paper, we assessed whether a citizen science program (*SoilSkin*) could be a viable option to increase the current knowledge of Biological Soil Covers from the Iberian Peninsula. Using a smartphone app (*eBryoSoil*) to gather data with the help of the public, we were able to build models to assess the response of BSCs to climate in five different habitat types, and thus, to infer their vulnerability. We identified two environmental variables, precipitation of the warmest quarter and the minimum temperature of the coldest month, that have a significant effect on BSC (lichens and bryophytes) cover. Our results also highlight the potential vulnerability of BSCs in habitats where they are more exposed to harsh conditions, such as those with less canopy cover (see Tabe 3). BSCs are small but crucial groups of organisms that have often been overlooked, despite their significant impact on ecosystem functioning. This has resulted in important gaps in the knowledge of their distribution and environmental dependencies^51^. Nonetheless, our results emphasize that the data gathered by citizen scientists can significantly increase our knowledge of Iberian Peninsula BSCs. This paves the way for the use of citizen science projects to study these small soil-dwelling communities.

### Public participation

Our citizen science program does not have the level of participation and public engagement of unstructured popular projects such as iNaturalist (which, for instance, has that gathered 3,602,949 observations within the same period of time in the Iberian Peninsula). Thus, it is necessary to contextualize our achievements under the light of our scope and purpose. With this paper, we show that the *eBryoSoil* app, which is part of a structured project that focuses on less-known organisms often overlooked in citizen science projects, has succeeded in reaching its on-going goals in its first stages of operation, in a niche area. The program gathered the attention of up to 800 participants in only 14 months.

Regarding participant engagement in the app, within the period of time used for this article, 142 of the participants registered in the application, 40 (28.2%) have uploaded records that were considered valid. Moreover, the observed temporal trend of increasing user engagement over time is promising, indicating a growing interest and potential for future contributions to the app. Despite these seemingly modest numbers, the significance of our study lies in its ability to engage a dedicated community of enthusiasts and researchers in a small but specialized field. BSCs present several challenges as targets for citizen science programs. These communities are complex, formed by small organisms that are difficult to identify and are mostly not known for the general public^52^. Thus, most people are unaware of their ubiquity, importance and beauty. The achieved level of engagement highlights the value and public interest in biodiversity segments that are frequently underrepresented in broader citizen science initiatives and shows that once the public is shown the importance and interest of these organisms, they are keen to engage in their study.

It is also worth noting the impact of the SARS-CoV-2 has had on our citizen science project. Like many other citizen science projects (see^53–55^), *SoilSkin* was heavily affected by the pandemic. Participation in the project reached a plateau at the end of the first quarter of 2020 (see Fig. 1), that was only possible to overcome when the confinement and other pandemic safety measures were lifted in the last quarter of 2020. Indeed, citizen science projects that depended on data collected in areas away from the home of participants received comparatively less participation during this period than projects that could be done at or near home^56^. This trend led to the additional undesirable consequence of perpetuating the positive survey bias towards the recorders’ home range (i.e., records are scattered and clustered, see, for example^36^), in detriment of locations like natural parks that are favoured in informal surveys by professional and amateur naturalists^57–59^. However, this shift also brings to light the resilience and adaptability of citizen science projects. Our experience with the *SoilSkin* project during this period aligns with trends observed in other citizen science initiatives, where engagement was challenged yet continued in varied capacities. Furthermore, the *SoilSkin* workshops, primarily conducted in public schools, played a pivotal role in promoting public participation in the project. The pandemic-induced drop in participation, especially from educators and students, mirrors the broader challenges faced by community science projects during this period. Nonetheless, the continued involvement of a dedicated group of participants, despite these challenges, highlights the robustness of our initiative and the enduring interest in specialized biodiversity research.

Regarding the validity of the records, there is a noticeable difference between the total of records and the ones that were considered as valid (see Fig. 1). Indeed, the identification of the species present in BSCs can be a difficult task for citizen scientists and other variables can affect the accuracy of the identification by the users (for instance the phenotypic variation of the BSCs may lead to not so accurate identification; light conditions of the sampling area; density of the BSCs). However, to be part of this citizen science project one is only required to identify the broad morphological type of each BSC constituent, which can be considered as an accessible taxonomic category. This simplification of protocols has already been suggested as a powerful tool to achieve higher success^60^. Our strategy, combining volunteer training and expert validation, has yielded high-quality distribution information for challenging study groups^61^.

Thus, paring a low taxonomic requirement with the training from workshops, we were able to accomplish a robust database that allowed us to improve our understanding of the potential impacts of climate change on the Iberian Peninsula BSCs. In conclusion, the *eBryoSoil* app’s success should be measured not just by the quantity of participation but by its quality and the specific niche it fills in biodiversity research. Its focus on underrepresented organisms in citizen science underscores its unique contribution to the field. The growing trend of engagement, despite external challenges, points to a bright future for this app and for similar initiatives focused on specialized ecological studies.

### The influence of environmental variables and habitat on BSCs

Our findings highlight the pivotal role of habitat on lichen and bryophyte cover. Specifically, our results suggest an association between the cover of these soil communities and densely forested areas (see Table 2). These areas, characterized by greater levels of canopy cover, can be associated with optimal moisture levels, supporting the persistence of these organisms^62,63^. In contrast, sparse or absent canopies expose BSCs to harsh climatic conditions, leading to lower cover in semi-arid regions with scarce rainfall, such as the shrublands^64^. Curiously, records gathered in grasslands seem to have a higher probability of containing BSC cover than the ones in open forest habitats. This might be the result of species interactions going in opposite directions depending on the abiotic conditions. While lichens and bryophytes often tend to engage in competitive interactions with vascular plants^65,66^, vascular plants in grassland habitats may also facilitate the presence of BSC through the regulation of microclimate conditions^67^. Regardless of the mechanism involved, our results highlight the fundamental importance of both forest and grassland habitats in the preservation of BSC communities. In particular, these findings stress the need for a better preservation of grassland habitats, which have traditionally been underappreciated despite their pivotal importance in ecosystem services and the biodiversity that provides them^68,69^.

Besides, climatic conditions (in particular, precipitation of the warmest quarter and the minimum temperature of the coldest month) emerge as critical factors influencing the persistence and abundance of lichens and bryophytes, which is consistent with the physiology of these organisms. Both groups are poikilohydric organisms, so their water status is completely dependent on the immediate conditions of their surrounding environment (see^70,71^). Notably, the precipitation in the warmest quarter plays a crucial role, as higher temperatures lead to greater evaporation rates. This results in shorter periods of photosynthetic activity and faster desiccation rates, which ultimately affects the abundance and growth rates of both bryophytes^21,72,73^and lichens^74,75^. For similar reasons, the significant effect of the minimum temperature of the coldest month in the variation of the cover of lichens and bryophytes is also explained by the high relevance of temperature controlling the growth rate of these poikilohydric organisms^21,73,75^.

Furthermore, interactions between habitat and climate variables have been shown to influence the persistence of lichens and bryophytes. For instance, a significant interaction between shrubland and precipitation of the warmest quarter correlates with higher abundances of lichens and bryophytes, as the rainfall helps the BSCs withstand the harsh conditions. This is elucidated by the fact that localities within the Mediterranean region are characterized by large periods without precipitation on the warmest months^76^. Our results indicate that grassland habitats in colder areas are more likely to have records with lichens and/or bryophytes. Additionally, we found that the absence of lichens and bryophytes is more probable in grassland habitats with higher minimum temperatures during the coldest month and increased precipitation in the warmest quarter. The fact that the presence of lichens and bryophytes is constrained by low temperatures in grassland habitats is surprising, considering how BSCs are well-adapted to withstand relatively low temperatures and are capable of maintaining high rates of photosynthetic productivity under such conditions^77,78^. However, the presence of continental areas suggests that the cold winter conditions in these regions could indeed limit BSC activity^79–81^. If this is the case, it is possible that an increase in temperature associated with climate change might favor an expansion of BSC cover in those areas. This unexpected interaction may also be explained by the constraints that extreme climate conditions (such as snow and ice) impose on vascular plants, which often facilitate the presence of BSCs. This again underscores the importance of the preservation of grassland habitats, which seem to be very susceptible to changes in climate^69^.

### Concluding remarks

Overall, the results obtained through data uploaded by citizen scientists show that it is possible to assess a connection between BSCs and the climatic factors to which they are exposed in several habitats of the Iberian Peninsula. This has important implications because it makes it possible to use such data for conservation strategies. According to our results, habitat seems to have the most decisive influence on the distribution and abundance of lichen and bryophyte cover. Furthermore, the effect of habitat was also amplified when considering the interaction with climatic variables. Thus, conservation efforts that aim to preserve habitats most favorable to the presence of BSCs is essential. In this context, it is important to preserve shrublands and grasslands, habitats that are seldom the focal point of conservation efforts. Thus, protecting such habitats not only safeguards biodiversity but also contributes to the overall health and functioning of ecosystems.

The results obtained throughout this citizen science project indicate that the information provided by the users of our specifically tailored citizen-science app can be useful for the characterization and distribution of BSC communities and thus to assess their vulnerability to climate change. This is despite the barriers imposed by the SARS-CoV-2 outbreak, which certainly hampered data gathering during most of the *SoilSkin* program. In conclusion, this program can be considered a successful example of how citizen science may be used to identify vulnerable habitats and regions in large territories, thus providing a blueprint for the design of future citizen science-based studies focusing on understudied and neglected organisms and/or communities.

## Data Availability

The datasets analysed during the current study are deposited in Zenodo, available through the following hyperlink: https://doi.org/10.5281/zenodo.4456773.
